# Modified arthroscopic repair of a posterior cruciate ligament tibial avulsion fracture improves IKDC and Lysholm score compared to open reduction

**DOI:** 10.1186/s13018-024-04851-4

**Published:** 2024-06-18

**Authors:** Xingxing Li, Qiming Ma, Quan Zheng, Qiangbing Dou, Liang Zhou, Liangye Sun, Song Shao, Qiwei Wang

**Affiliations:** 1 Department of Orthopedics, Lu’an Hospital of Anhui Medical University, Lu’an, 237001 China; 2 Department of Orthopedics, The First Affiliated Hospital of Anhui Medical University, Hefei, 230022 China; 3 Anhui Medical University, Hefei, 230022 China

**Keywords:** Tibial avulsion fracture, PCL, Arthroscopy, ORIF, Treatment outcome

## Abstract

**Purpose:**

The purpose of this study was to analyse the difference between arthroscopic fixation and open reduction internal fixation (ORIF) of posterior cruciate ligament (PCL) tibial avulsion fractures.

**Methods:**

This retrospective study analysed patients with an acute PCL tibial avulsion fracture who underwent surgical treatment at our hospital and follow-up for at least 24 months. Variables based on sex, age, Meyers–McKeever type, surgical method, meniscus tear, external fixation, labour or sports, Lysholm knee score, IKDC score, and KT-1000 value were also recorded. Multifactor unconditional logistic regression and Student’s t test with 1:1 propensity score matching (PSM) to remove confounding factors were used for analysis.

**Results:**

Sixty-five cases achieved knee function graded as “good” or better, and 9 cases not. Single-factor analysis indicated that Meyers–McKeever type (*χ*^2^ = 4.669, *P* = 0.031) and surgical approach (*χ*^2^ = 9.428, *P* = 0.002) are related to functional outcomes. Multifactorial logistic regression analysis further confirmed that Meyers–McKeever typing (OR = 10.763, *P* = 0.036, [95% CI 1.174–98.693]) and surgical approach (OR = 9.274, *P* = 0.008, [95% CI 1.794–47.934]) are independent risk factors affecting prognosis. In addition, PSM verified significant differences in the Lysholm score (t = 3.195, *P* = 0.006), IKDC score (t = 4.703, *P* = 0.000) and A-KT/H-KT (t = 2.859, *P* = 0.012). However, the affected-side KT-1000 value (A-KT, mm, t = 1.225, *P* = 0.239) and healthy-side KT-1000 value (H-KT, mm, t = 1.436, *P* = 0.172) did not significantly differ between the two groups. The proportions of cases in which the Lysholm score, IKDC and A-KT/H-KT exceeded the minimal clinically important difference (MCID) were 62.5% (20/32), 62.5% (20/32) and 93.75% (30/32), respectively.

**Conclusion:**

Compared with ORIF, an arthroscopic approach for PCL tibial avulsion fractures achieves better results.

*Level of evidence*: Retrospective cohort study; Level II.

**Supplementary Information:**

The online version contains supplementary material available at 10.1186/s13018-024-04851-4.

## Introduction

The incidence of PCL system (including tibial avulsion, femur avulsion and ligament) injuries is lower than that of the anterior cruciate ligament (ACL) system [8, 14, 26]. As the diameter of the PCL is thicker than that of the ACL, the proportion of tibial avulsion fractures to ligament ruptures is higher [17]. Avulsion fracture of the PCL is a kind of knee joint injury that can promote knee joint instability accelerate the long-term degeneration of the knee joint [3, 5]. Avulsion fracture of the tibia is usually caused by high-energy injuries during kneeling on something hard, which are common in motorcycle accidents [1, 7].

PCL tibial avulsion fractures were first reported in 1975 [18], and the need for surgery is recognized by orthopaedic surgeons [6]. Radiograph can provide valuable information to assist clinical diagnosis [27]. PCL reconstruction was to be a potential alternative surgical method due to the improvement of in bone tunnel fabrication, graft replacement, and graft reinforcement [20–23, 33]. Compared to tendon to bone healing, bone to bone healing was faster and more reliable [25], and fracture reduction internal fixation were more easily accepted. Arthroscopic approaches and ORIF are equally satisfactory [7, 13, 31]; however, some scholars have suggested that the outcomes of the two operations are unequal [24, 29]. This may be due to differences in surgical approach or the method of arthroscopic internal fixation [34]. As the low incidence and the small number of cases [14, 30], few data are available to compare outcomes of open versus arthroscopic repairs from the same centre. In Asia, the incidence of this fracture has increased significantly with the use of electric bicycles [10], and we aim to investigate the impact of these two methods on outcomes. This study is based on a modified arthroscopic method. The purpose of this study was to analyse the difference between arthroscopic fixation and ORIF for PCL tibial avulsion fractures. We hypothesized that the arthroscopic approach would result in better outcomes than ORIF.

## Methods

### Patient enrolment

**Fig. 1:**
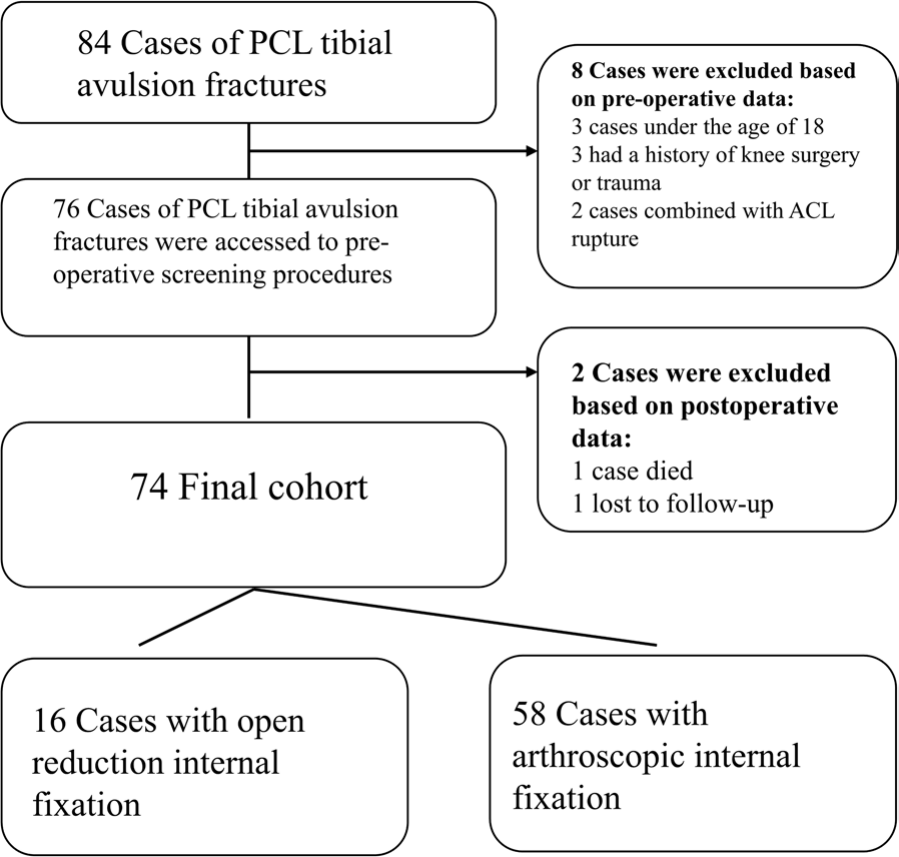
Flowchart of the patient selection process

The ethical review committee at our hospital approved the study, and informed consent was obtained from all participants. The study was performed according to the Declaration of Helsinki. Patients hospitalized between March 2018 and April 2021 were eligible. Case inclusion criteria were as follows: (1) acute PCL tibial avulsion fracture (< 3 weeks), (2) normal joint prior to injury, (3) fracture types were Meyers–McKeever typing II, III or IV, and (4) ability to understand the medical staff and to cooperate. Exclusion criteria were as followed: (1) combined repair with other ligamentous system injuries or fractures (e.g., ACL, collateral ligament, tibial plateau fracture), (2) failure to follow the prescribed functional training after surgery, as documented in the subsequent outpatient medical records, (3) neuromuscular disease (due to potential risk of impact on joint function), and (4) age < 18 years old (Fig. 1). 76 patients eligible for this retrospective cohort study were divided into arthroscopic and ORIF groups. The cases in this study were from an ongoing prospective study with a follow-up period of no less than 2 years. Grouping conditions were determined by hospitalization ID, with odd numbers being the arthroscopy group and even numbers being the ORIF group.

Prior to the final interview, patient information was retrieved from the case management system. The basic information collected was follows: sex, age, Meyers–McKeever typing [19], surgical approach, meniscal tear, postoperative external fixation, and whether the patient participated in labour activities or sports prior to the injury (Table 1). Patients were invited to our hospital for a final exam, and two physicians (who were not involved in data compilation and statistics) were responsible for collecting functional information (including Lysholm Knee Score, International Knee Documentation Committee [IKDC] subjective score [4], KT-1000 value and 2 or 3 of exclusion criteria). Patients were unaware of the group to which they were assigned.

**Table 1:** Baseline characteristics and univariate analysis

Related factors		Lysholm ≥ 85 (n = 65)	Lysholm < 85 (n = 9)	*χ*^2^	*P* value
Gender	Male	35	6	0.135^a^	0.713
	Female	30	3		
Age	< 40 years	30	4	0.000^a^	1.000
	≥ 40 years	35	5		
Meyers–McKeever typing	II–III	40	1	4.669^a^	*0.031*
	IV	25	8		
Surgical approach	ORIF	10	6	9.428^a^	*0.002*
	Arthroscopy	55	3		
Meniscus tear	Yes	29	7	2.279^a^	0.131
	No	36	2		
External fixation	Yes	30	6	0.637^a^	0.425
	No	35	3		
Sports or labor before injury	Yes	40	4	0.380^a^	0.537
	No	25	5		

Italic values represent the differences in the statistically significant

^a^Continuous corrected chi-square test was used when the minimum expected value was between 1 and 5

### Surgical technique

The operations were performed under general anaesthesia, and all patients received antibiotics 30 min before the operation. ORIF patients were placed in the prone position, and arthroscopic approach patients were positioned supine on the operating table with a tourniquet tied to the root of the thigh with the pressure set at 60 kPa. The intersection of the tourniquet and the skin of the thigh was closed with iodophor film, and a sterile waterproof cavity towel was placed on the distal part of the tourniquet after disinfection.

ORIF was performed with a traditional posterior S-shaped incision. Entering through the space between the medial head of the gastrocnemius and the semimembranosus, the gastrocnemius was pulled laterally along with the popliteal vascular nerve. At the level of the joint capsule, an incision was made at its attachment to the posterior tibial cortex. The fracture end was cleaned with physiological/normal saline. Hollow screws (Supplementary Fig. 1) or anchors with sutures (Supplementary Fig. 2) were chosen for fixation according to whether the fracture was simple or comminuted. At the end of the case, a deep drain was placed adjacent to the bone surface.

**Fig. 2:**
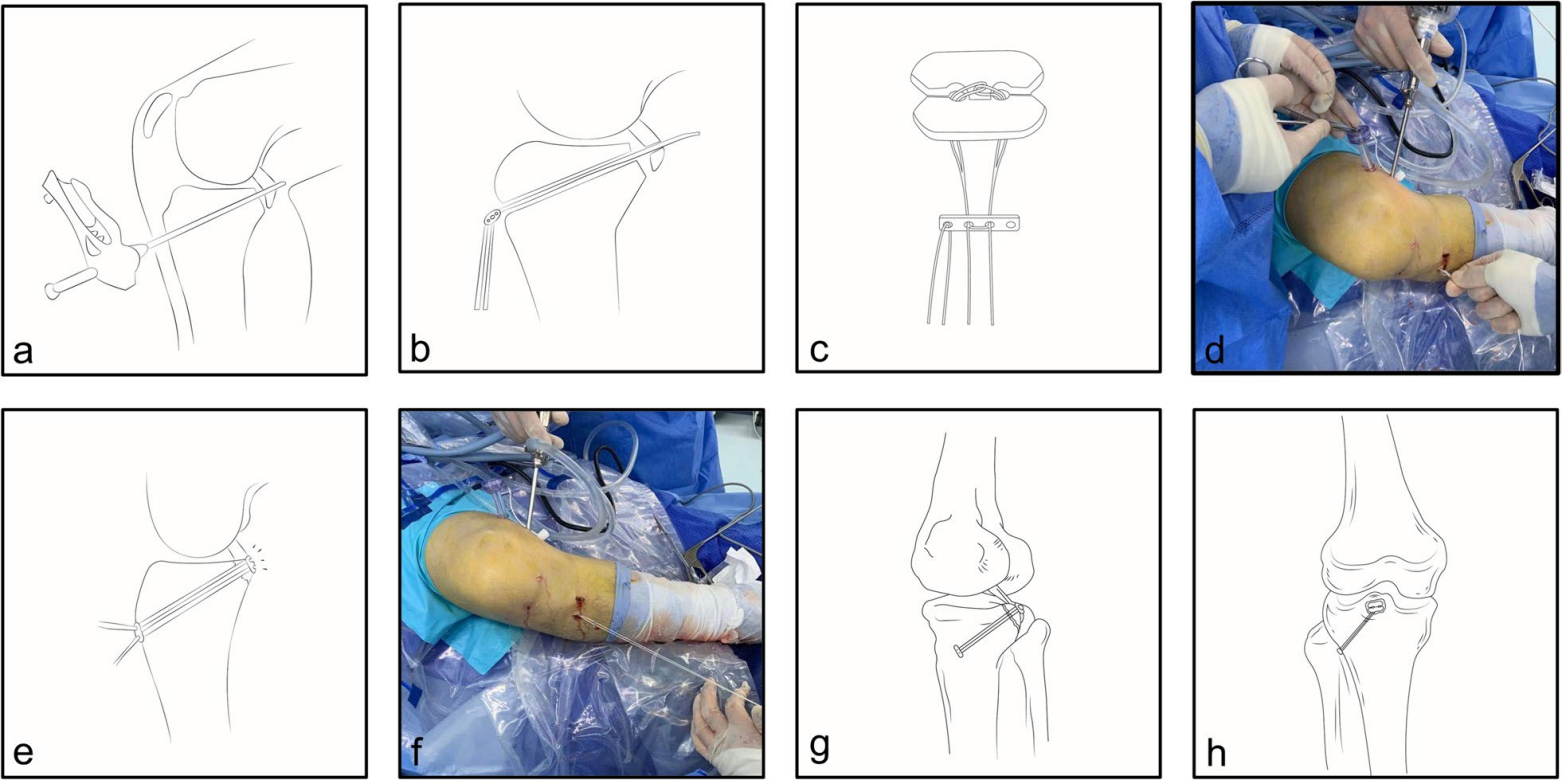
Diagram of arthroscopic reduction internal fixation technique

The arthroscopic procedure began with an anteromedial and anterolateral standard approach to perform a diagnostic arthroscopy (Supplementary Fig. 3). The torn meniscus was treated with suturing or shaving. With the knee flexed and hip externally rotated ("4" test), posteromedial and superior approaches were used for visualization and manipulation, respectively, and an 8-gauge working cannula was implanted using the superior approach (Supplementary Fig. 4). To expose the fracture, the synovial membrane and part of the joint capsule behind the PCL was removed, and a clean and properly deepened bone bed was essential for repositioning and restoring PCL tension. A 2.0-mm diameter Kirschner needle (fine needle to avoid fragmentation of the fracture) was inserted in the upper 1/3 of the fracture fragment from the anterior tibia using an ACL point sight in the reset condition (Fig. 2a). A No. 2 PDS wire was inserted into the joint cavity along the bone tract through a 16-gauge puncture needle, and the suspension plate wire loop (ACL TightRope RT with Titanium and UHMWPE, Arthrex, United States) was passed anteriorly and posteriorly through the bone block to the joint cavity (Fig. 2b), where it was pulled outside the body by a working cannula. A “工” titanium plate (TightRope, ABS, Button, 8 × 12 mm, Arthrex, United States) was attached outside the body (Fig. 2c), returned to the articular cavity along the trocar (Fig. 2d) and placed on the surface of the fracture block (Fig. 2e), with the fracture block compressed (Fig. 2g, h) and the wire loop tightened anteriorly (Fig. 2f). Refer to supplementary video 1 for a comprehensive overview of the arthroscopic procedure. No drainage tube was placed for the procedure.

Figure 2 showing bone tunnel was made (a), TightRope plate was introduced (b), ABS was connected (c), ABS was implanted into the joint (d), fracture block was pressurized (e), TightRope was tightened (f), and finally the lateral (g) and anteroposterior (h) position were performed. The black arrow points to TightRope ABS Button. The white arrow points to ACL TightRope RT with Titanium and UHMWPE.

**Fig. 3:**
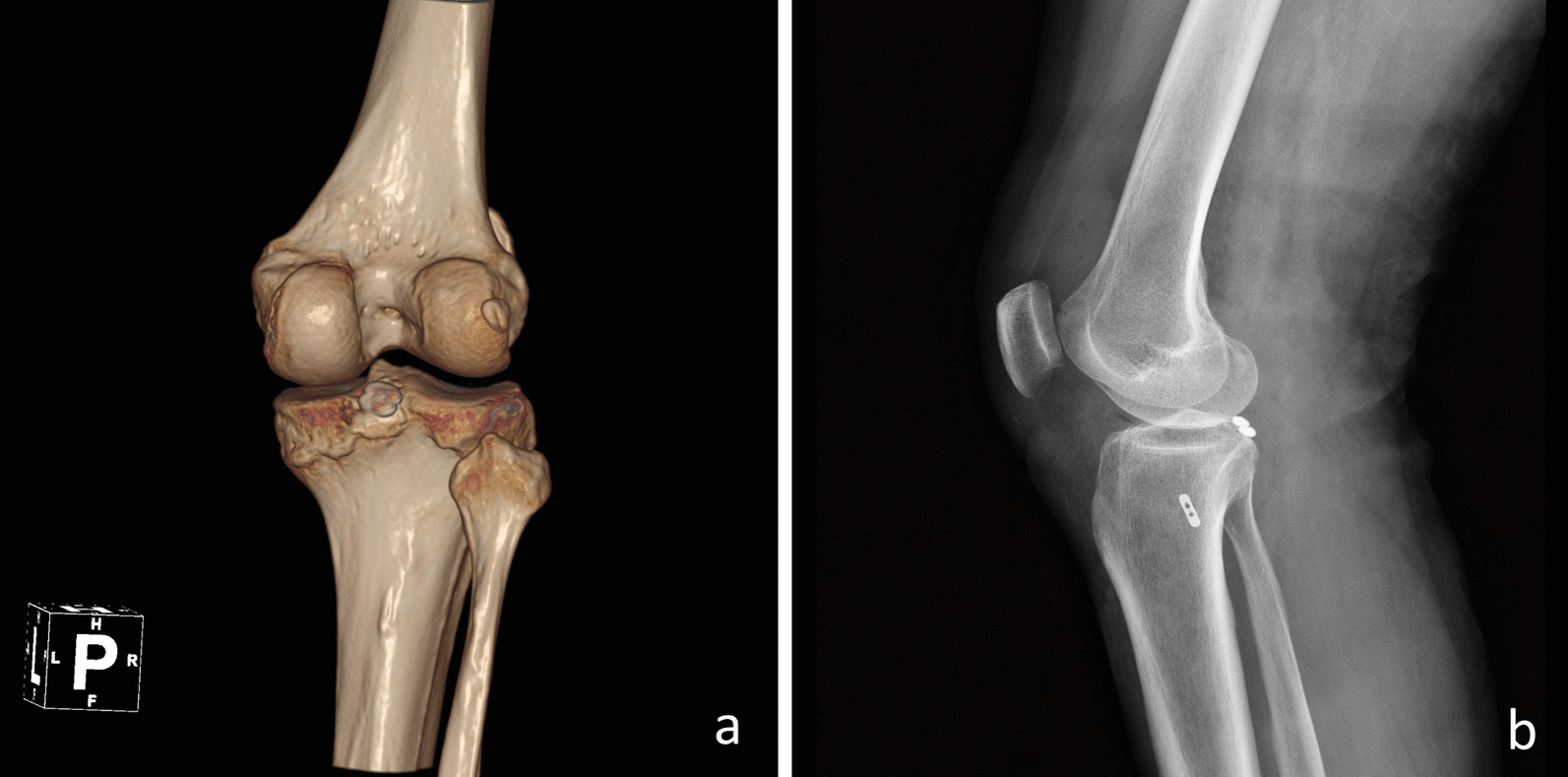
Arthroscopic case

After fixation, the stability of the fracture was tested by flexion and extension of the knee joint. In addition, intraoperative X-rays were used to evaluate whether the procedure was satisfactory. Antibiotics were administered for 48 h after surgery, and low molecular weight heparin was used for routine anticoagulation. A representative example is presented in Fig. 3. Supplementary Figs. 5 to 7 demonstrate knee function and incision healing at the 7-week postoperative review.

Figure 3 showing Day 2 postoperative 3D CT (a, Posterior) and Day 2 postoperative X-ray (b, lateral position) of a 53-year-old male patient operated on using arthroscopy.

### Postoperative rehabilitation

On postoperative Day 1, the patient was asked to perform at least 300 quadriceps contractions and ankle pumps. To avoid blood buildup in the joint cavity, knee flexion exercises were not performed for 3 days after surgery. Then, patients were allowed to determine whether they underwent external fixation with a hinged brace after signing the informed consent form, but knee flexion exercises were required. The postoperative range of motion was 0–60° at 2–4 weeks, 0–90° at 4–6 weeks, 0–120° at 6–8 weeks, and full range of motion after 8 weeks postoperatively. Ambulation without a brace was allowed at 6 weeks provided that the patient could stand alone on the affected limb to demonstrate adequate muscle strength.

### Data analysis

All data were processed using SPSS 25.0 and RStudio 3.6.8. Univariate analysis was performed using the chi-square test or Fisher’s exact probability method as appropriate. The model was based on whether the patient was older than 40 years old (youth ≤ 40, middle-aged > 40) and whether the fracture was type IV. Logistic multiple regression was used to analyse risk factors affecting the outcome. Surgical modality was used as a grouping condition, fracture type and meniscal tear were used as screening conditions, the calliper was set to 0.02, and confounding factors were removed using 1:1 PSM. Student's t test for paired samples was used for the final analysis to further clarify differences in the efficacy of the two procedures. MCID was calculated as ½ SD of the delta. A *P* value < 0.05 was considered a statistically significant difference.

## Result

All eligible cases were enrolled in the study. The 10 excluded cases (11.90%) included three cases with a history of surgical or traumatic knee experience (one case in the arthroscopic group and two cases in the ORIF group), three cases of patients < 18 years old (ORIF group), two cases with comorbid ACL rupture (ORIF group), one case who died 3 weeks after discharge (ORIF group), and one case who was lost to follow-up (arthroscopic group 1, 1.19%). Of the remaining 74 patients (41 men and 33 women), 58 underwent arthroscopy, and 16 underwent ORIF, with an average follow-up time of 35.27 ± 8.88 months (24–57 months) and an average age of 43.23 ± 0.36) years (20–69 years). Causes of injury were as follows: 35 cases of motorized two-wheeler accidents (arthroscopic Group 28, ORIF Group 7), 17 cases involving falling from a height (arthroscopic Group 9, ORIF Group 6), 13 sports injuries (11 cases in the arthroscopic group, 2 in the ORIF group), and 9 injuries were sustained while descending steps (8 in the arthroscopic group, 1 in the ORIF group). The mean days from trauma to operation was 5.32 ± 1.99 (days). The mean operative time was 71.16 ± 10.5145 (min). There were no postoperative complications, including infection, nonunion of the fracture, and failure of internal fixation requiring secondary surgical removal.

The results of the univariate analysis identified Meyers–McKeever (*χ*^2^ = 4.669, *P* = 0.031) and surgical approach (*χ*^2^ = 9.428, *P* = 0.002) as factors potentially affecting surgical outcomes (Table 1). Using a Lysholm score ≥ 85 as the criterion for efficacy determination [16], a logistic multiple regression analysis of several variable potentially affecting treatment efficacy revealed that Meyers–McKeever typing (OR = 10.763, *P* = 0.036, [95% CI 1.174–98.693]) and surgical approach (OR = 9.274, *P* = 0.008, [95% CI 1.794–47.934]) were independent risk factors (Table 2). Subgroup analysis results show that ARIF was better than either hollow screw (OR = 0.025, *P* = 0.001, [95% CI 0.003–0.213]) or anchor fixation (OR = 0.073, *P* = 0.028, [95% CI 0.007–0.756]) in improving joint functions.

**Table 2:** Multi-factor logistic regression

Related factors	β	S. E	Wald	df	Sig	Exp(B)	95% CI of Exp(B)	
							Lower	Upper
Constant	− .164	.609	.072	1	.788	.849	–	–
Surgical approach	2.227	.838	7.062	1	.008	9.274	1.794	47.934
Meyers–McKeever type	2.376	1.131	4.417	1	.036	10.763	1.174	98.693

Sixteen pairs of comparable data were obtained by PSM. Analysis of the data indicated that operative time (70.56 ± 10.91 [min] vs 71.75 ± 10.42 [min]), *P* = 0.766), affected side KT-1000 (4.875 ± 2.557 [mm] vs 5.25 ± 2.739 [mm]), *P* = 0.239) and healthy side KT-1000 (2.088 ± 0.761 [mm] vs 1.938 ± 0.7679 [mm], *P* = 0.172) did not differ between the two groups. However, VAS score for incision pain (4.063 ± 1.289) vs 5.438 ± 1.459, *P* = 0.008), Lysholm score ( 96 (interquartile rang (IQR), 91 to 97) vs 87.5 (IQR, 82 to 92.25), *P* = 0.019), IKDC (96.5 (IQR, 93 to 98.25) vs 87.5 (IQR, 81 to 90.25),* P* = 0.002), and A-KT/H-KT (1.3 (IQR, 1.1 to 1.92) vs 4 (IQR, 2.95 to 4.9),* P* < 0.001) exhibited statistically significant differences (Table 3).

**Table 3:** Characteristics of patients underwent arthroscopic approach and ORIF

Variables	Arthroscopy (n = 16)	ORIF (n = 16)	*P* value
Sex, n (%)^a^			0.031
Male	6 (38)	13 (81)	
Female	10 (62)	3 (19)	
Age (years), median (Q1, Q3)^b^	47.5 (33, 52.25)	48.5 (35.5, 55.25)	0.806
Cause of injury, n (%)^a^			0.235
Electric bicycle accident	7 (44)	7 (44)	
Falling from height	2 (12)	6 (38)	
Sports injury	6 (38)	2 (12)	
Step down accident	1 (6)	1 (6)	
Days of hospitalization (days), median (Q1, Q3)^b^	8 (6.75, 9.25)	8 (7, 11.25)	0.285
Days from trauma to operation (days), Median (Q1, Q3)^b^	5.00 (4.00,6.00)	5.50 (4.00,6.25)	0.848
Mean of operative time, minutes (SD)^c^	70.56 (10.91)	71.75 (10.42)	0.766
Meyers McKeever type, n (%)^a^			1
II	1 (6)	2 (12)	
III	5 (31)	4 (25)	
IV	10 (62)	10 (62)	
Meniscal tears, n (%)^a^			1
No	6 (38)	6 (38)	
Yes	10 (62)	10 (62)	
External fixation, n (%)^a^			0.722
No	10 (62)	8 (50)	
Yes	6 (38)	8 (50)	
Labour or sports, n (%)^a^			1
No	5 (31)	5 (31)	
Yes	11 (69)	11 (69)	
Follow up time (Months), Median (Q1, Q3)^b^	31.5 (25.5, 40.25)	38 (26, 45.25)	0.335
Mean VAS score for incision pain, (SD)*^c^	4.063 (1.289)	5.438 (1.459)	0.008
Lysholm scores, Median (Q1, Q3)^b^	96 (91, 97)	87.5 (82, 92.25)	0.019
IKDC score, Median (Q1, Q3)^b^	96.5 (93, 98.25)	87.5 (81, 90.25)	0.002
Mean of Affected side KT-1000 A-KT, mm (SD)^c^	4.875 (2.557)	5.25 (2.739)	0.239
Mean of Healthy side KT-1000 H-KT, mm (SD)^c^	2.088 (0.761)	1.938 (0.767)	0.172
A-KT/H-KT, Median (Q1, Q3)^b^	1.3 (1.1, 1.92)	4 (2.95, 4.9)	< 0.001
MCID, n (%)^a^			1
Lysholm score	10 (62.5)	10 (62.5)	
IKDC	10 (62.5)	10 (62.5)	
A-KT/H-KT	15 (93.75)	15 (93.75)	

IKDC, International Knee Documentation Committee; A-KT, Affected-side KT-1000 value; H-KT, Healthy-side KT-1000 value; SD, Standard deviation; VAS, Visual analogue scale; MCID, Minimal clinically important difference; Q1, Lower quartile; Q3, Upper quartile

*Scores on the VASs for incision pain range from 0 to 10, with higher scores indicating more severe pain

^a^Chi-squared test with Yates’ continuity correction

^b^Fisher’s exact test

^c^Independent-samples t-test

## Discussion

Our study demonstrated that the arthroscopic approach is superior to ORIF for PCL tibial avulsion fractures. The most common mechanism underlying PCL avulsion fractures of the tibia is dashboard collision, in which a direct force is applied to the proximal part of the tibia in an anterior-to-posterior direction with the knee in flexion [31]. The rarity of PCL tibial avulsion fractures makes the comparison of the efficacy of the two surgical approaches challenging to study [2]. In the cohort, we compared differences in joint function and related factors after the two surgical procedures. Although random allocation may avoid selection bias to a certain extent, it may bring about class imbalance. More significantly, due to the limited number of cases, the power of this research is limited and the potential for type II error is relatively high, although we balanced the data by PSM.

Our analysis demonstrated that sex, age, meniscus tear, postoperative assisted external fixation made with a brace, and previous participation in sports or physical activity did not significantly affecting prognosis. We had initially predicted that the presence of a meniscal tear would be associated with differences in treatment outcomes, but the results were inconsistent with our expectations. In addition, postoperative external fixation was not mandatory. Results following customary postoperative management may differ [36, 37]. Because refusal to employ external fixation does not affect outcomes, the strength of the internal fixation modalities we selected was adequate and did not cause displacement of the fracture fragment without external fixation protection. Participation in sports or physical activity before the injury was considered a factors that may affect the treatment outcome because such individuals have greater muscle strength, which may be advantageous later rehabilitation [15]. The results suggest that quadriceps strength training with 300 or more leg lifts per day is fully adequate for normal walking after 6 weeks.

PCL avulsion injuries were fixed with hollow screws or sutured fixation with wire anchors, and some studies have demonstrated that the fixation strength of these two methods is adequate [6, 7, 11]. The drawbacks to an open approach include a long and unsightly incision, pain from compression of the incision during knee flexion, and surgical interference with neurovascular structures, which are the primary reasons for the transition to an arthroscopic approach [7]. It is noteworthy that in the cases we excluded, a 28-year-old male patient died of pulmonary embolism due to deep vein thrombosis of the lower limbs in the third week after surgery. As this patient underwent open reduction, the risk of vascular interference should be considered.

Our arthroscopic approach used a familiar technique, with slight modifications to make it more widely applicable. Compared with open reconstruction, this surgical approach can also be used for PCL reconstruction to reduce trauma and the potential risk of damage to popliteal blood vessels [28]. First, we used 2.0-mm/2.4-mm diameter Kirschner pins for bone tunnel drilling to avoid excessive bone loss or fragmentation of the fracture block due to the use of 4.5 mm drills [9, 32]. The 2.0-mm Kirschner pins better ensured the integrity of the fracture block, but the aiming accuracy was not as ideal as the 2.4-mm Kirschner pins. If the isolated bone mass is destroyed, the fracture is upgraded to Meyers–McKeever type IV. According to our findings, the prognosis of type IV (comminuted fracture) fractures was worse than that of type II or III (isolated fracture) fractures, and the difficulty of arthroscopic fixation of the fragmented bone mass was increased. We did not analyse the correlation between fracture type and various outcome measures (IKDC or KT-1000), but the results indicate that fracture type was independently associated with a Lysholm score ≥ 85 (achieved knee function graded as “good” or “excellent” [9, 32]). Considering the poorer fixation strength of a comminuted fracture compared to a simple fracture, it is possible that peripheral bone fragments of a comminuted fracture may detach from the bone bed under ligamentous pull, resulting in a loss of tension in the ligament to which the fragment is attached. However, this is not the case with a simple fracture because there is only one piece of bone. A toothed plate may be more stable [3] but requires open surgery. In addition, some studies have reported the use of the TightRope plate to compress the bone block inferiorly [12, 35, 37]. We believe that the TightRope plate has too little coverage of the fracture block, tends to compress the fracture block into two or more flaps, and cannot be effectively fixed for a comminuted bone block. We used the ABS plate under compression fixation, which not only avoided the problem of stress concentration in the fracture block but also fixed Meyers–McKeever type IV fractures. The fixation was achieved by simply placing the ligamentous bone into the depression (not seeking to reset all the fragments) and covering it with ABS, as the ABS was sufficiently large to cover the entire fracture bed. The final compression phase was performed in front of the tibia, and we used the TightRope to avoid the problem of loose wire knots, which conduce to closer approximation of the bone block and fracture bed and improved fracture stability to improve healing. During the attachment to ABS, the original structure of the TightRope is used, and there is no need for any additional attachments. This ensures that the fixation represents a complete system to avoid loosening or separating the two parts during compression or rehabilitation exercises.

Interestingly, knee stability assessment revealed no difference in KT-1000 between the affected and healthy sides. There was a statistically significant difference in the of A-KT/H-KT ratio, suggesting that the original stability should be considered when evaluating knee stability.

## Conclusion

The surgical approach as well as fracture type were independent risk factors for the outcome of PCL tibial avulsion fractures. Compared with ORIF, an arthroscopic approach for PCL tibial avulsion fractures achieves better results. ABS plate repair provides a new reference for the minimally invasive repair of intra-knee fractures and has great prospects for clinical promotion.

## Limitations

This study had several shortcomings. First, it was a retrospective study, and further high-level studies are needed to support the findings. Second, we were unable to follow up each case regularly or to capture the differences between the two groups during the recovery process. Furthermore, the duration of follow-up was inconsistent between cases, especially for ORIF, which may have impacted the final results. Additionally, we did not distinguish fracture type as an independent influencing factor when including cases, which may affect the results. Finally, there was insufficient biomechanical evidence to verify the strength of the ABS plates for fixation of the fracture blocks.

### Supplementary Information

Supplementary Material 1.

Supplementary Material 2.

## Data Availability

The datasets used and/or analyzed during the current study are available from the corresponding author on reasonable request.

## Abbreviations

PCL: Posterior cruciate ligament
ACL: Anterior cruciate ligament
ARIF: Arthroscopic reduction internal fixation
ORIF: Open reduction internal fixation
IKDC: International Knee Documentation Committee
PSM: Propensity score matching
A-KT: Affected-side KT-1000 value
H-KT: Healthy-side KT-1000 value
MCID: Minimal clinically important difference
VAS: Visual analogue scale
SD: Standard deviation
IQR: Interquartile rang
Q1: Lower quartile
Q3: Upper quartile
