# Supplementary material for: Modified arthroscopic repair of a posterior cruciate ligament tibial avulsion fracture improves IKDC and Lysholm score compared to open reduction
Source: J Orthop Surg Res. 2024 Jun 18;19:362. doi: 10.1186/s13018-024-04851-4 (PMC11184816; doi:10.1186/s13018-024-04851-4)

Supplementary figure 1: A lateral X-ray of knee the second day after surgery fixed by hollow screws


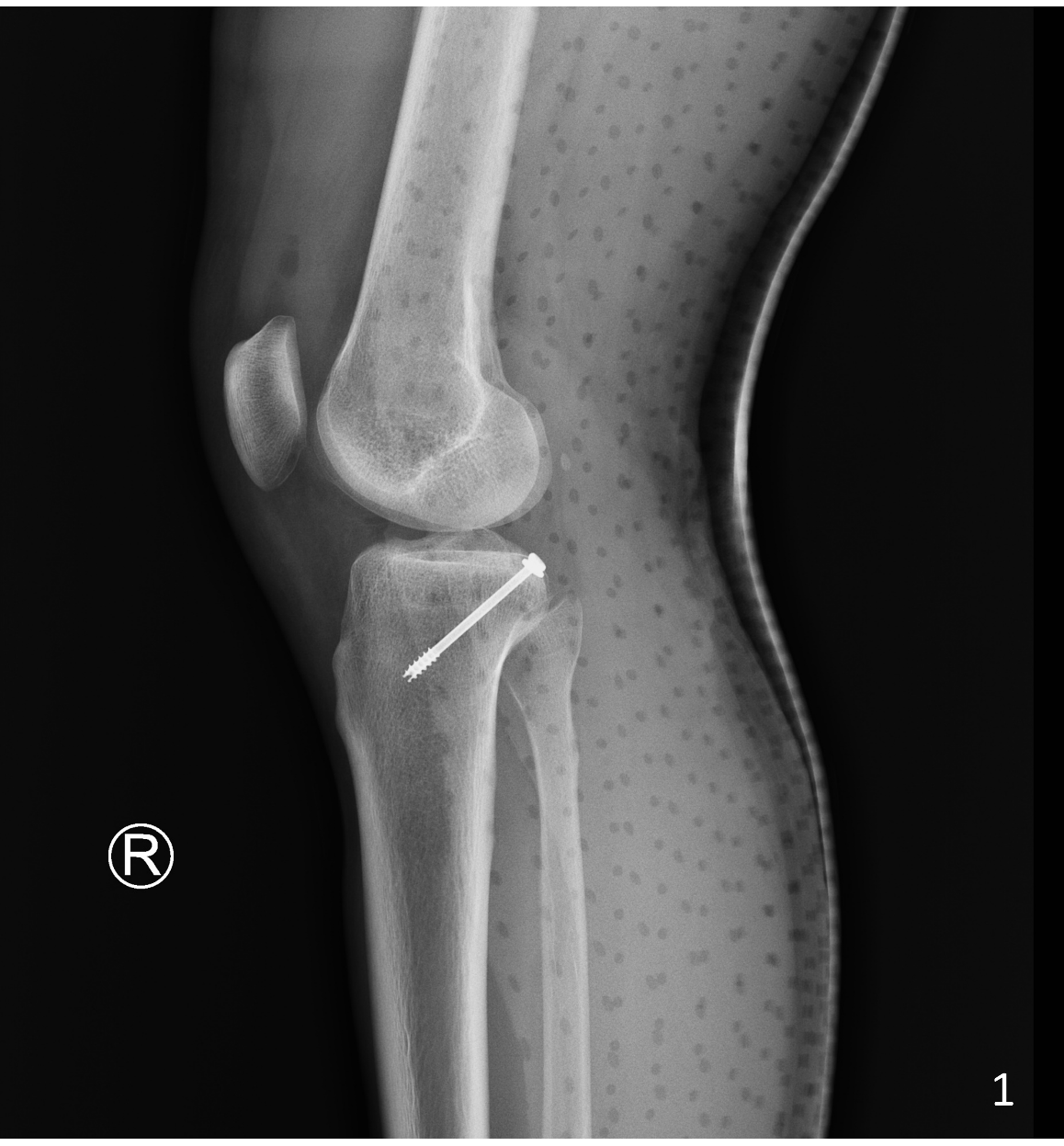


Supplementary figure 2: A lateral X-ray of knee the second day after surgery fixed by anchors with sutures


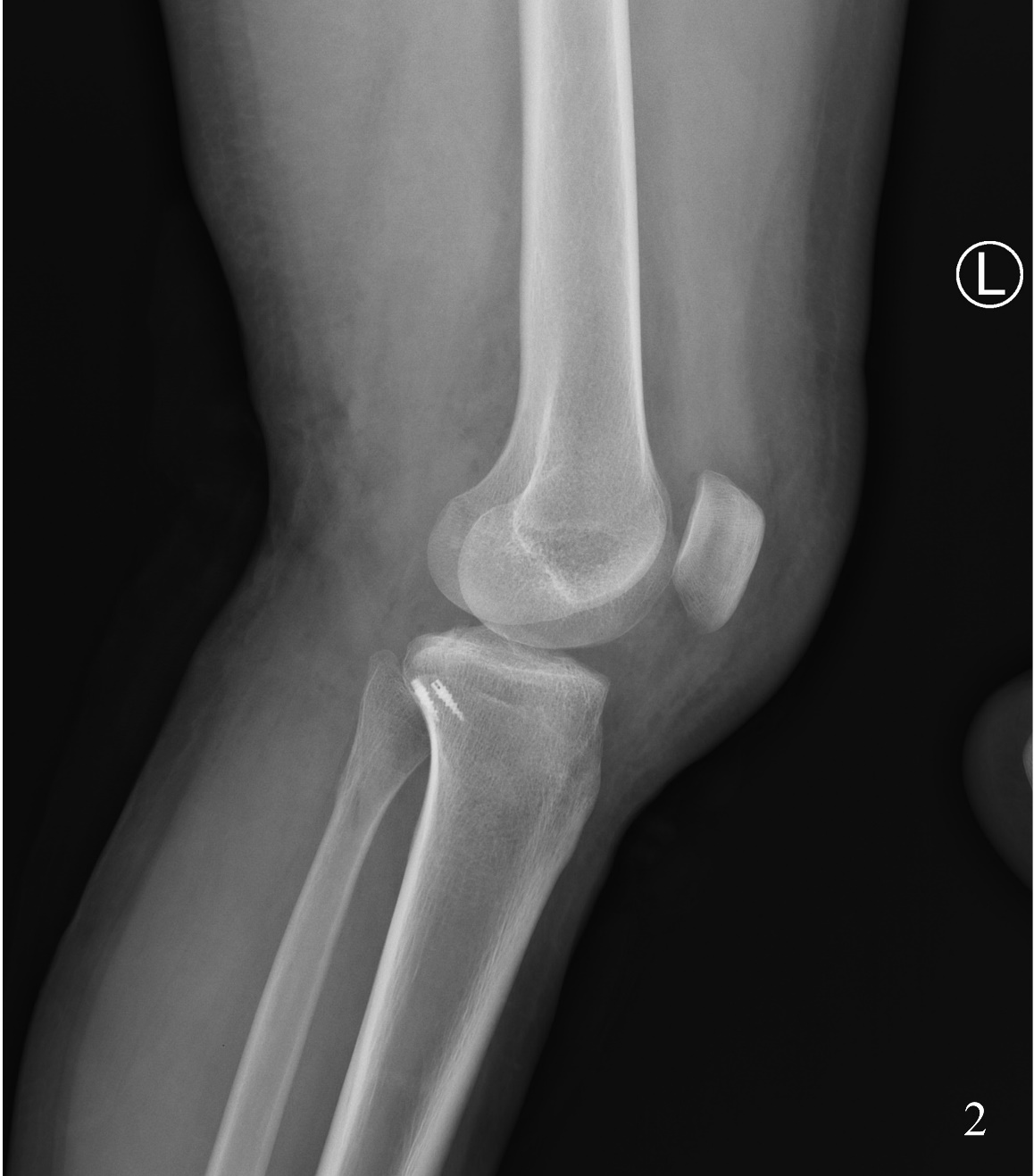


Supplementary figure 3: surgical incision(anterior view)


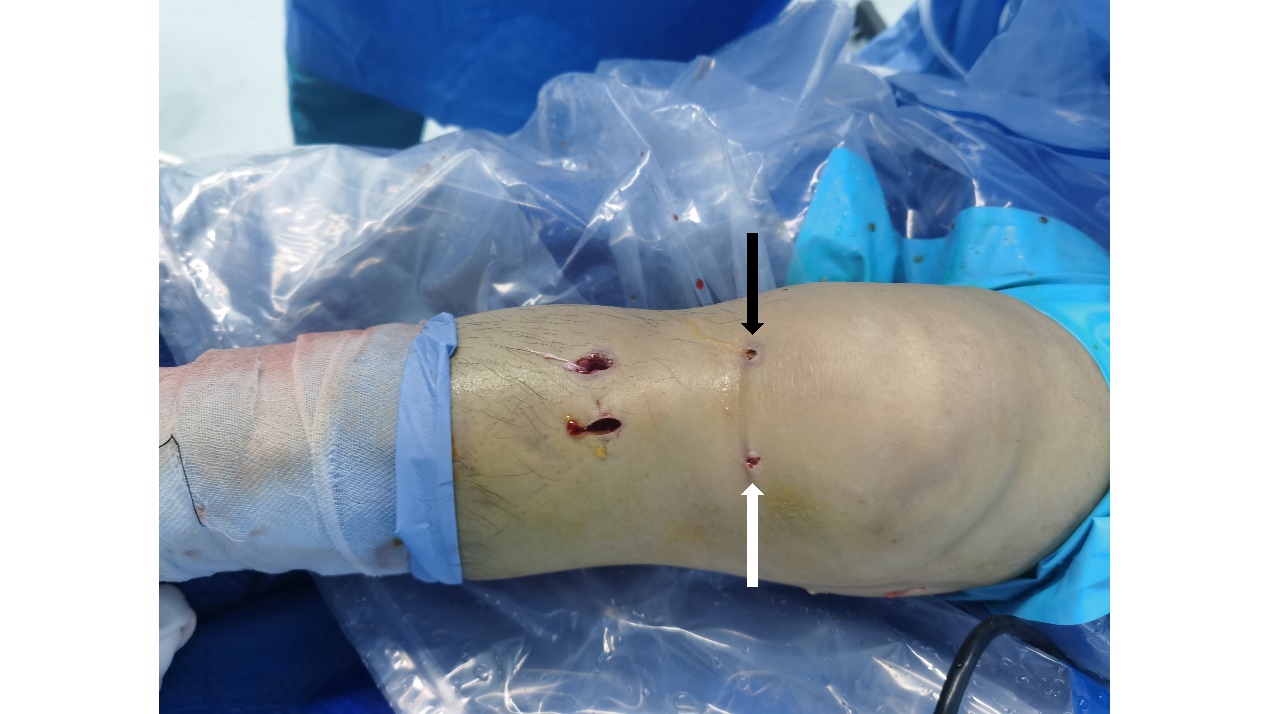


The black arrow points to anterolateral approach. The white arrow points to anteromedial approach.

Supplementary figure 4: surgical incision(interior view)


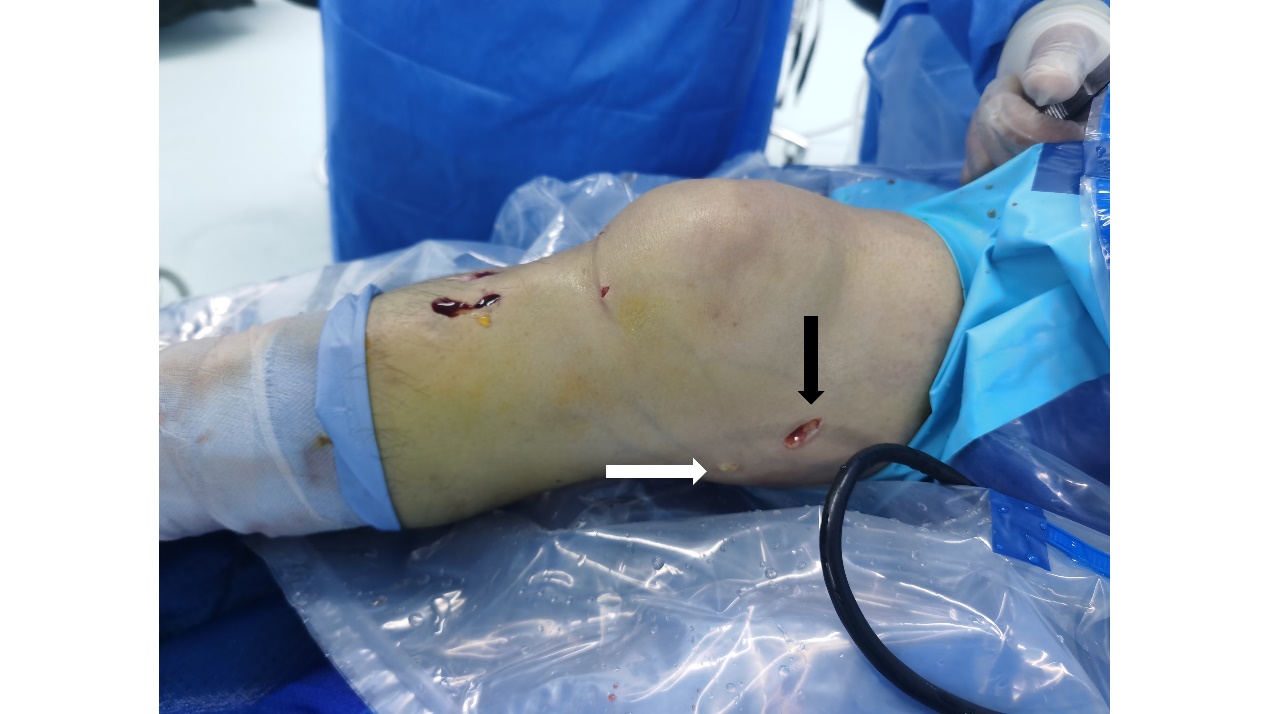


The black arrow points to posterior high approach. The white arrow points to posterior inferior approach.

Supplementary figure 5: Knee flexion up to 120° seven weeks after surgery (external view)


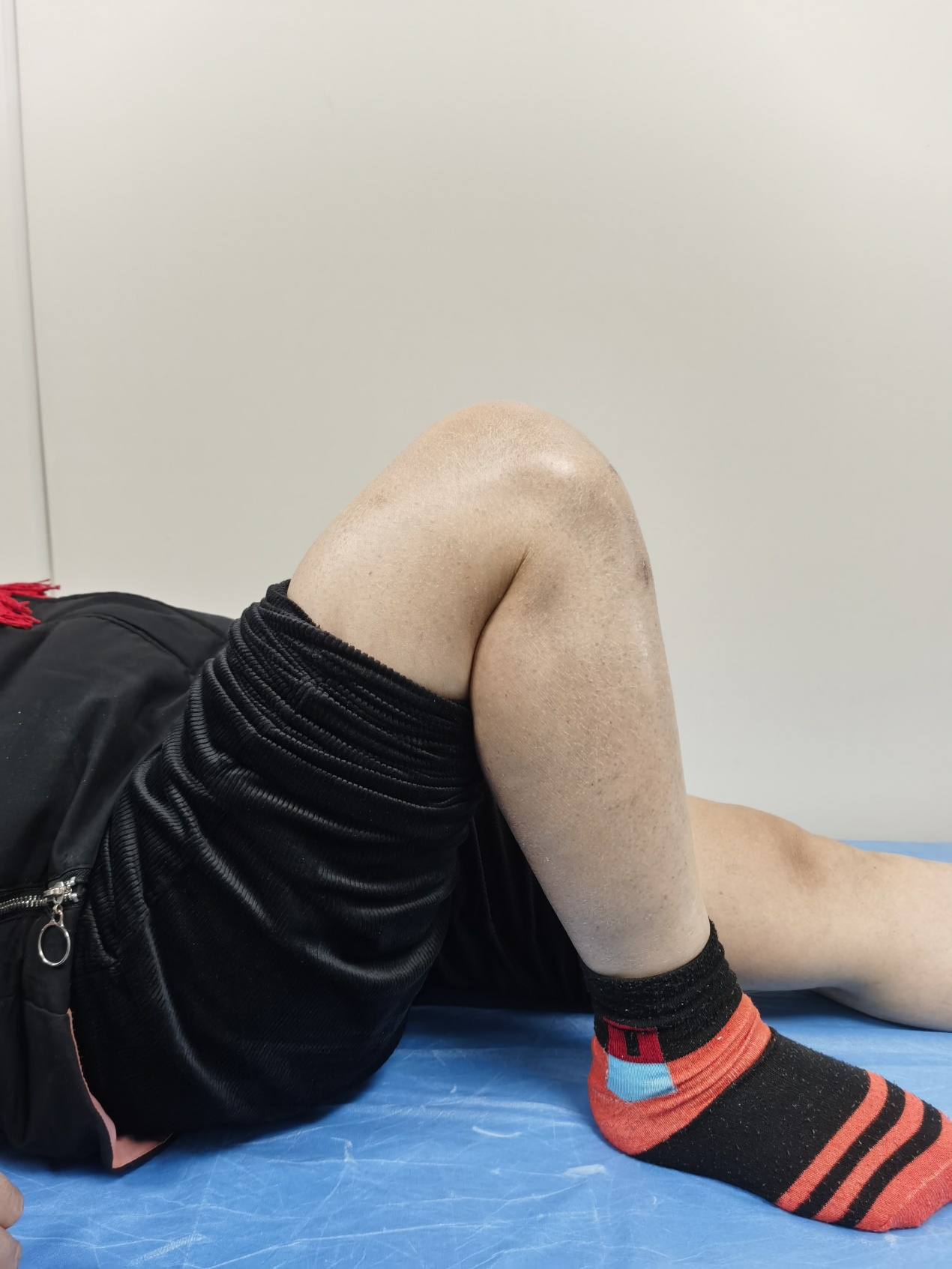


Supplementary figure 6: The incision has completely healed(interior view)


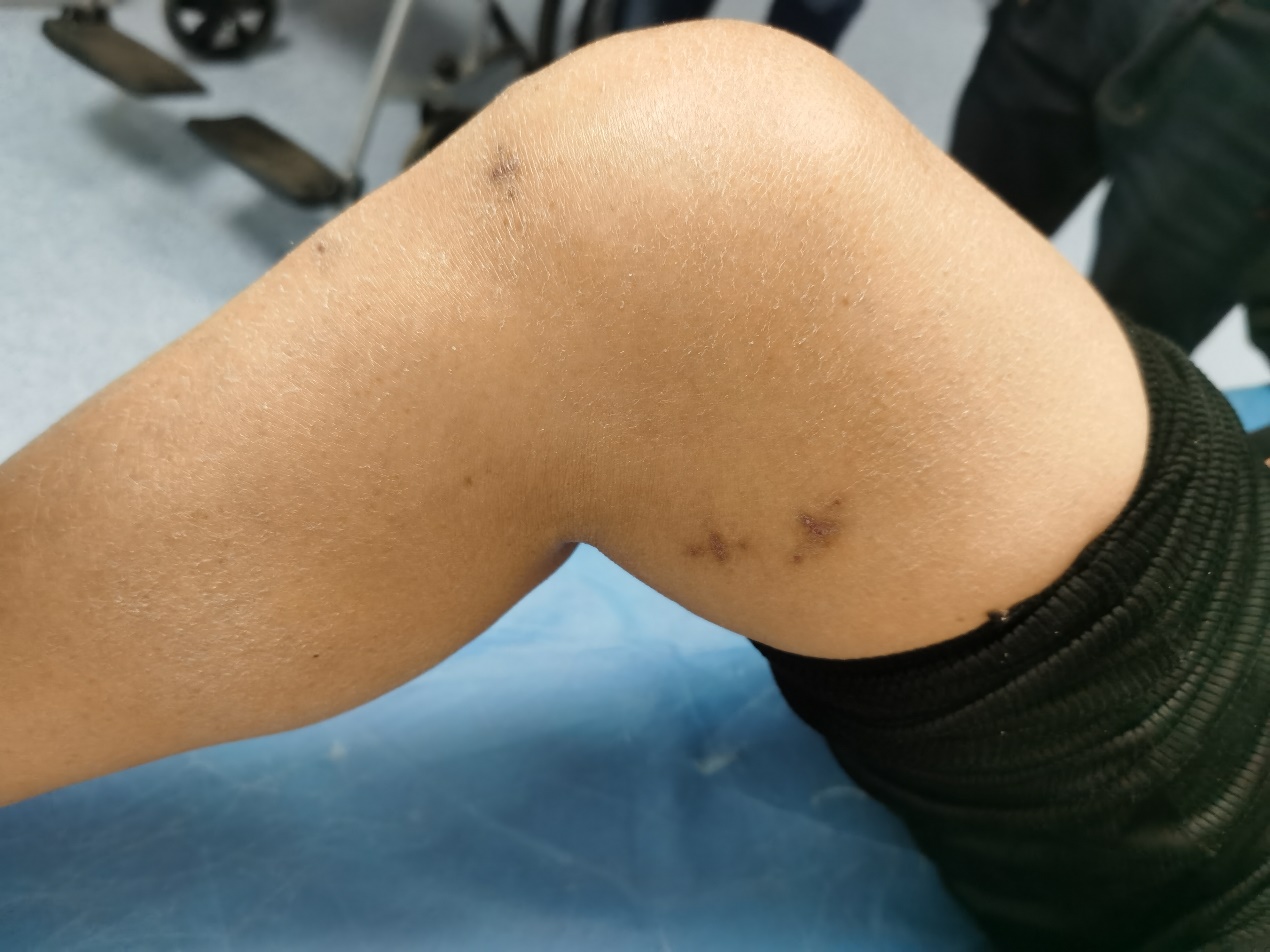


Supplementary figure 7: The knee joint can be fully extended(anterior view)


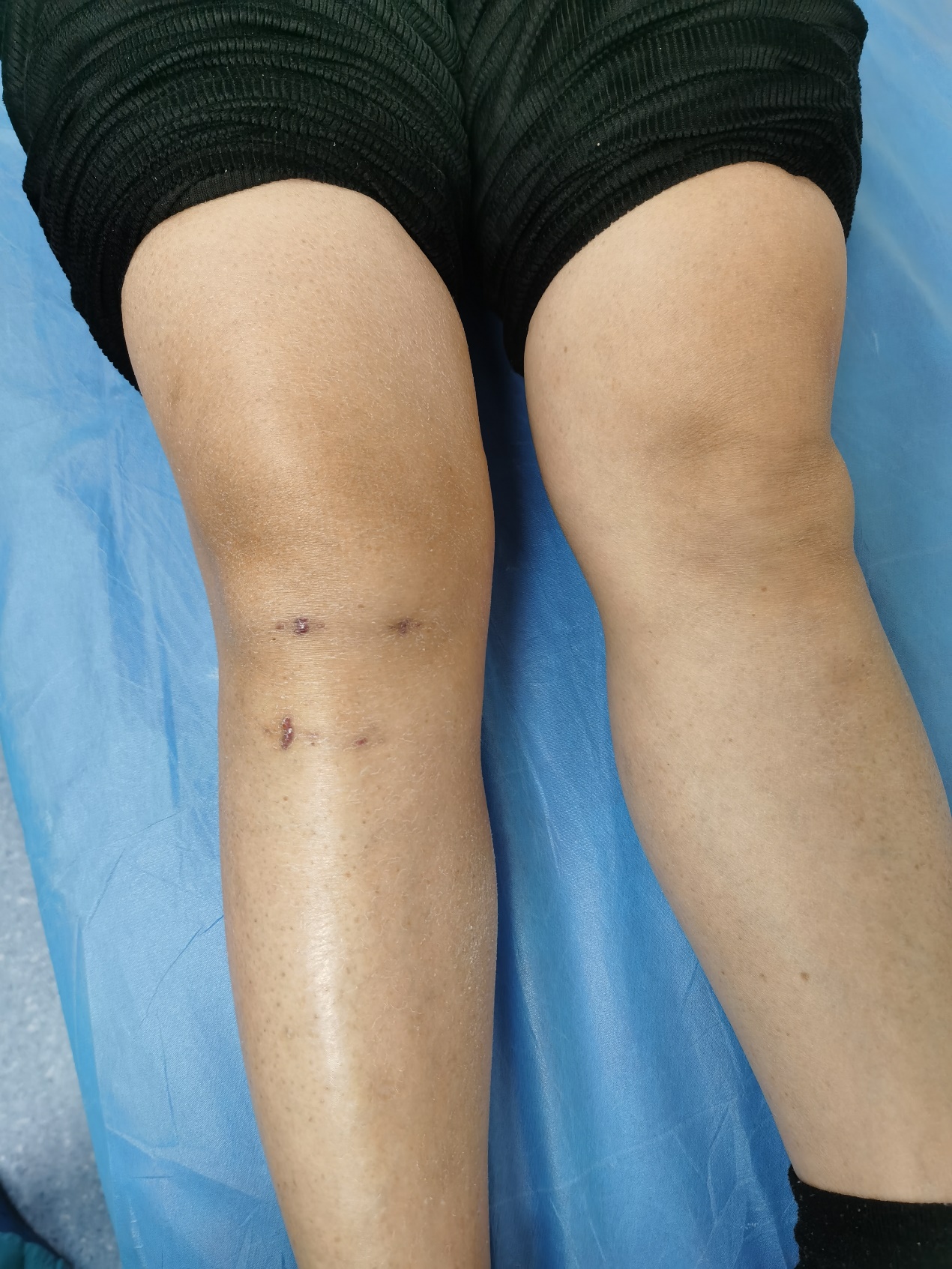

Supplement: Supplementary file 1 — Supplementary Material 1. [file 13018_2024_4851_MOESM1_ESM.docx]
